# Au@Ag@Pt Trimetallic Nanoparticle-Enhanced Dual-Mode Lateral Flow Immunochromatographic Assay for Ultrasensitive and Rapid Detection of Acetamiprid in Plant-Based Foods

**DOI:** 10.3390/foods15152598

**Published:** 2026-07-24

**Authors:** Qingqing Li, Xuecheng Zhang, Shurun Chen, Bowen Guo, Qingwen Cao, Xinyue Feng, Xiaoping Yu, Xiaoyu Jia, Biao Zhang, Hongqing Wang

**Affiliations:** 1 Key Laboratory of Microbiological Metrology, Measurement & Bio-Product Quality Security, State Administration for Market Regulation, College of Life Sciences, China Jiliang University, Hangzhou 310018, China; liqingqing7295@163.com (Q.L.); 2200901111@cjlu.edu.cn (X.Z.); 19057243957@139.com (S.C.); guobowen2024@163.com (B.G.); fxy18767053369@163.com (X.F.); yxp@cjlu.edu.cn (X.Y.); 2 Hangzhou Institute of Food and Drug Control, Hangzhou 310022, China; cqw891002@hotmail.com; 3 Institute of Agricultural Products Preservation and Processing Science and Technology, Academy of Agricultural Sciences, Tianjin 300384, China; jiaxiaoyu331@163.com

**Keywords:** acetamiprid, Au@Ag@Pt, lateral flow immunochromatography assay, rapid detection, plant-based food

## Abstract

Acetamiprid (ACE) is a widely used neonicotinoid insecticide with potential health risks due to its persistence in the environment and bioaccumulation in plant-based foods. In this study, an improved dual-mode lateral flow immunochromatography assay (DMLFIA) was established for the sensitive detection of ACE residues in plant-based foods using Au@Ag@Pt NPs as dual-function signal probes. In the colorimetric mode (Mode 1), with the inhibition rate as the vertical coordinate and the ACE concentrations as the horizontal coordinate, the linear detection range of the colorimetric mode was 0.1–3000 μg/L with the detection limit of 0.08 μg/L. In the photothermal mode (Mode 2), the temperature values obtained via smartphones integrated thermal imager showed a linear relationship with 0.1–3000 μg/L of ACE, and the detection limit is 0.1 μg/L. The cross-reactivity rate of the developed DMLFIA with other structural analogs and functional substances was less than 0.39%. The recovery rate of the developed DMLFIA used in spiked plant-based foods was 81.1–99.4%, with relative standard deviations (RSD) of 1.3–10.7%. The detection results of this constructed DMLFIA showed good consistency with those obtained by HPLC and ELISA. The constructed DMLFIA provides a selective, sensitive and rapid detection tool for ACE in different scenarios in plant-based foods.

## 1. Introduction

Neonicotinoid insecticides have emerged as the most extensively used class of insecticides worldwide owing to their high insecticidal efficiency [1], low mammalian toxicity, and systemic properties [2]. Acetamiprid (ACE), as a representative first-generation neonicotinoid, acts on nicotinic acetylcholine receptors (nAChRs) of insects, blocking neurotransmission and causing paralysis and death of target pests [3,4]. It is extensively used in the cultivation of lily, corn, apple, and other crops to control aphids and sucking pests [5,6]. Nevertheless, ACE exhibits long environmental half-life and resistance to conventional degradation, leading to excessive accumulation in agricultural commodities [7]. Long-term intake of plant-based foods containing excessive ACE residues may induce neurotoxicity and pose chronic health risks to humans [8,9]. Therefore, sensitive and rapid detection of ACE residues is urgently required.

To safeguard public health, China has established the national standard GB 2763-2021 [10], specifying maximum residue limits (MRLs) for ACE in plant-based foods. At present, mainstream analytical techniques for ACE residues mainly include instrumental methods such as high-performance liquid chromatography (HPLC), liquid chromatography-tandem mass spectrometry (LC-MS/MS), gas chromatography-mass spectrometry (GC-MS/MS), and so on [11,12,13]. These methods feature high accuracy and sensitivity, but require expensive instruments, professional operators, and complicated sample pretreatment processes, which are not conducive to high-throughput screening and on-site rapid detection [14,15]. Therefore, it is necessary to develop new high-sensitivity rapid detection methods.

Enzyme-linked immunosorbent assay (ELISA) is one of the most widely used conventional immunoanalytical techniques, and its detection performance is highly dependent on signal probes [16,17]. As a classical signal tag, traditional colloidal gold suffers from low signal intensity and a narrow linear range, which hinders the improvement of immunoassay detection sensitivity [18,19]. To break through the inherent limitations of conventional probes, advanced nanomaterials have been continuously developed for biosensing applications [20,21,22]. Among them, multimetallic nanoparticles show great potential due to tunable optical properties, large specific surface area, and high stability [23,24,25]. In particular, Au@Ag@Pt NPs integrate the strong localized surface plasmon resonance (LSPR) of Au/Ag and the high photothermal activity of Pt, rendering them outstanding signal amplification candidates for high-efficiency immunoassays [26,27,28].

In this study, Au@Ag@Pt NPs with excellent photothermal properties were synthesized and followed by conjugation with ACE monoclonal antibodies for signal probes. Critical experimental parameters, such as coating concentrations of test and control lines, buffer pH value, surfactant content, probe dosage and immunoreaction duration were systematically optimized to enhance detection capability. Accordingly, a dual-mode lateral flow immunochromatography assay (DMLFIA) was established for ACE residue detection. In addition, the specificity of the DMLFIA was evaluated using non-target pesticides, and its accuracy and reliability were validated by comparison with conventional analytical methods including HPLC and ic-ELISA. This DMLFIA possesses dual-mode detection characteristics, allowing flexible selection of detection modes for diverse application scenarios and achieving mutual verification of detection results.

## 2. Materials and Methods

### 2.1. Materials and Reagents

Acetamiprid (ACE), atrazine, dinotefuran, nitenpyram, chlorpyrifos, imidacloprid, thiacloprid, and trichlorfon were purchased from Shanghai Aladdin Biochemical Technology Co., Ltd. (Shanghai, China). Chloroauric acid (HAuCl_4_), β-mercaptopropionic acid, bovine serum albumin (BSA), N-hydroxysuccinimide (NHS), 1-ethyl-(3-dimethylaminopropyl) carbonyldiimide, K_2_PtCl_4_, and hydrochloride (EDC) were purchased from Sigma-Aldrich (Shanghai, China). Dimethyl sulfoxide (DMSO), sodium carbonate (Na_2_CO_3_), sodium bicarbonate (NaHCO_3_), sodium chloride (NaCl), anhydrous magnesium sulfate (MgSO_4_), anhydrous sodium acetate (NaOAc), acetonitrile, methanol and sodium citrate were purchased from Shanghai Aladdin Biochemical Technology Co., Ltd. Tween-20 was obtained from Shanghai Macklin Biochemical Technology Co., Ltd. (Shanghai, China). Goat anti-mouse IgG was purchased from Jackson Immunoresearch Laboratories (West Grove, PA, USA). Monoclonal antibody of acetamiprid was purchased from Shenzhen KeJie industrial Co., Ltd. (Shenzhen, China). Nitrocellulose membranes (NC membranes), conjugate pads, absorbent pads, and PVC backing cards were purchased from Jinbiao Biotechnology (Shanghai, China). The goat anti-mouse IgG antibody was immobilized on the control line (C-line). The coating antigen ACE-BSA was coated on the test line (T-line). Ultrapure water was obtained from a Millipore Milli-Q system and used for the preparation of all solutions.

### 2.2. Instruments

A Milli-Q ultrapure water system was purchased from Millipore (Billerica, MA, USA). A Varioskan LUX multimode microplate reader (VLBL00D2) was purchased from ThermoFisher Scientific (Waltham, MA, USA). The 96-well EIA/RIA plates were purchased from Corning Incorporated (Corning, NY, USA). The Agilent 1100-Thermos TSQ Quantum Ultra was purchased from Thermo Fisher Scientific (Waltham, MA, USA). The DNP-9052B electric thermostatic incubator was purchased from Mingde Instrument (Hangzhou, China). The HM3035 XYZ three-dimensional film gold sprayer was purchased from Jinbiao Biotechnology (Shanghai, China). The BIO-VINOSTECH programmable single-knife cutting machine was purchased from Hangan Electronic Technology (Shanghai, China). The ultraviolet-visible spectrophotometer (UV-5200) was purchased from Metash instruments Co., Ltd. (Shanghai, China). A desktop high-speed centrifuge (CT1SRE) was purchased from Hitachi, Ltd. (Tokyo, Japan).

### 2.3. Preparation of Au@Ag@Pt NPs and Au@Ag@Pt-mAb

Au@Ag@Pt NPs were synthesized at room temperature via a mild citrate reduction method under precise control. Unless otherwise specified, concentrations are expressed as indicated. Briefly, 1.0 g trisodium citrate was dissolved in 100.0 mL deionized water under magnetic stirring at 800 r/min. The resulting solution was heated to 80 °C, followed by the addition of 10.0 mg solid silver nitrate, and the mixture was continuously stirred at the identical stirring speed for 30 min. The prepared Ag colloid had a total volume of 100 mL, and only 1.5 mL was used. Subsequently, only 1.5 mL of the prepared Ag colloidal suspension was transferred to a clean reaction vessel, and 1.0 μL of 10% HAuCl_4_ solution and 12.5 μL of 20 mmol/L K_2_PtCl_4_ solution were added. Upon complete reagent addition, the reaction vessel was tightly sealed, and the mixture was magnetically stirred at room temperature for 12 h. The as-prepared colloidal suspension was preserved at 4 °C for subsequent use. The 100.0 μL Au@Ag@Pt colloidal solution was mixed with 400.0 μL 0.01 mol/L PBS, and the pH of the mixture was adjusted to 8.0 with 0.2 mol/L K_2_CO_3_ solution. 10.0 μL acetamiprid antibody solution with a concentration of 3.7 mg/mL was added to the above solution, and the reaction was carried out at 4 °C for 4 h. After the reaction was completed, 5.0 μL PEG-20000 aqueous solution was added, and then 20.0 μL 20% (*w*/*v*) BSA aqueous solution was added, and the reaction was carried out at 25 °C for 1 h. Then the mixture was centrifuged at 4000 rpm for 15 min at 4 °C. The Au@Ag@Pt-mAb probe was obtained by washing twice with PBS and resuspended in 0.01 M PBS (1.0 mL, pH 6.0).

### 2.4. Preparation of Coating Antigen

Coating antigen (ACE-BSA) was prepared with minor modifications to previously reported methods. Synthesized ACE hapten (3.4 mg) was dissolved in 3.0 mL of ultrapure water. Aqueous solution containing 4.5 mg EDC and 3.0 mg NHS (300.0 μL) was added to the hapten solution. After 4 h activation at room temperature, the mixture was added dropwise to 5.0 mL BSA solution (10.0 mg/mL, 0.1 M NaHCO_3_, pH 8.3–8.5). Conjugation was carried out under gentle stirring at 4 °C for 12 h. Reaction mixture was dialyzed against PBS at 4 °C for 3 days, with dialysate renewed daily. Purified ACE-BSA conjugate was diluted to a final concentration of 6.5 mg/mL and stored at 4 °C for subsequent experiments.

### 2.5. Assembly and Parameter Optimization of Strips

The absorbent pad, NC membrane, and sample pad were sequentially pasted onto a PVC backing card. ACE-BSA coating antigen and goat anti-mouse IgG antibody were sprayed onto the NC membrane as the T-line and C-line, respectively, with a dispensing volume of 0.5 μL/cm. The assembled strips were dried at 37 °C overnight and cut into 3.96 mm widths, then stored dry with desiccant for later use. Different parameter conditions exert certain influences on the results of visual observation. Therefore, the parameters such as T-/C-line coating concentration (10–50 times dilution), buffer pH (5.0–10.0), Tween type and concentration (5–20%, Tween-20/Tween-80), probe volume (0.5–5.0 μL) and immune reaction time (5–25 min) were systematically optimized. The optimization criteria include clear bands, high T/C values, low background, and good repeatability. The prepared test strips were strictly stored under constant temperature conditions throughout the experiment.

### 2.6. Performance Evaluation of the Constructed DMLFIA

A series of ACE standard solutions with gradient concentrations (0, 0.1, 1.0, 10.0, 100.0, 500.0, 1000.0, 3000.0 μg/L) were prepared using PBS buffer (pH 6.0). For each test, 100 μL of the solution was dropped onto the sample pad, and the strip was incubated at room temperature for 20 min. The signal intensity of T-line and C-line was read by colloidal gold reader, and the inhibition rate was calculated. The inhibition rate was calculated as follows:

Inhibition rate (%) = 100 × (B_0_ − B)/B_0_

where B_0_ is the signal intensity of negative samples, and B represents the signal intensity of tested samples. Subsequently, the T-line photothermal signal was recorded under 808 nm laser irradiation and the temperature change (ΔT) was quantified. The visual limit of detection (vLOD) of DMLFIA is defined as the minimum concentration at which the T-line is significantly shallower than the C-line. The instrumental limit of detection (LOD) was defined as the concentration of acetamiprid corresponding to the inhibition rate of 10%. It was a universal quantitative detection limit index for food pesticide residue immunoassay, which could match the micro-detection requirements of the maximum residue limit of Chinese herbal medicines in GB 2763-2021. The corresponding concentration of 50% inhibition was used to compare the sensitivity of different immune methods.

For specificity evaluation, seven pesticides with similar structures or application scenarios, including atrazine, dinotefuran, nitenpyram, chlorpyrifos, imidacloprid, thiacloprid, and trichlorfon, were selected for cross-reactivity (CR) testing. Each pesticide was prepared into gradient solutions and tested using the optimized strip. The CR value was calculated as follows:

Cross-reactivity = IC_50 (ACE)_/IC_50 (Non-target pesticides)_ × 100%

For accuracy and precision assessment, six representative plant-based foods (tangerine peel, lily, fresh corn, soybean, apple and grape) without ACE confirmed by HPLC were used for standard addition recovery test. ACE standard solution was added to the samples at three concentration levels (50, 100, 200 μg/L). After optimizing the pretreatment (homogenization, extraction, centrifugation, filtration), the samples were measured three times in parallel. The recovery and relative standard deviation (RSD) were calculated to evaluate the accuracy and precision. By comparing with HPLC and ic-ELISA methods, the effectiveness of the method was further verified.

### 2.7. Pretreatment of Samples and Qualification of Instrument

Six actual samples-dried tangerine peel, dried lily bulb, fresh edible corn, soybean, apple, and grape, were subjected to pretreatment. Dried tangerine peel and lily bulb were crushed, sieved (40 mesh), and 2.0 g was extracted with 15 mL of 1% acetic acid-acetonitrile by vortexing (2 min) and ultrasonication (15–20 min). After salting-out with 6.0 g anhydrous MgSO_4_ and 1.5 g anhydrous NaOAc, the mixture was centrifuged (6000 rpm, 5 min), and the supernatant was filtered (0.22 μm) and diluted 10-fold with 0.01 mol/L PBS (pH 6.0). Soybean was ground, sieved, and 2.0 g was extracted with 15.0 mL acetonitrile, vortexed (3 min), and ultrasonicated for 20 min. Subsequent steps were identical to those for dried samples. Fresh corn, apple, and grape were homogenized, 2.0 g of the homogenate was extracted with 15.0 mL acetonitrile or 1% acetic acid-acetonitrile, vortexed (2 min), and ultrasonicated (10 min). After centrifugation and filtration, the extract was diluted 5-fold with PBS (pH 6.0) to mitigate matrix effects. For each test, 100.0 μL of the diluted extract was loaded onto the sample pad. After 20 min, T-line and C-line signals were recorded with a strip reader, and the T/C value was used for quantification. All samples were spiked at three levels (50, 100, 200 μg/L) and analyzed in triplicate. Results were compared with those obtained by HPLC and ic-ELISA. The specific steps of ELISA are shown in Supporting Information, and the gradient conditions of HPLC are shown in Table S1.

## 3. Results

### 3.1. Principle of the Established DMLFIA

**Figure 1:**
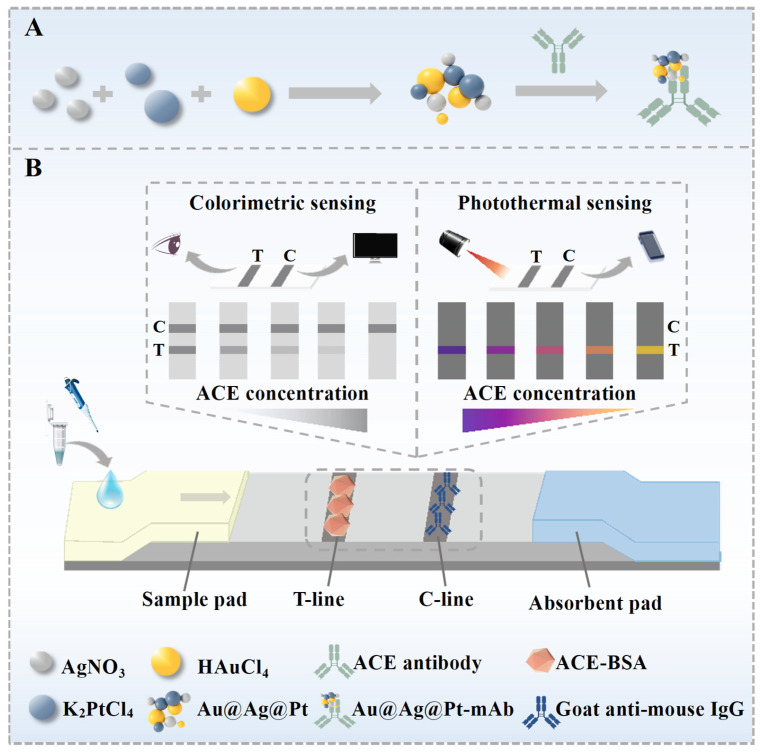
Schematic diagram of DMLFIA for detection of ACE. The synthesis route of Au@Ag@Pt-mAb (**A**), and the dual-mode detection output based on Au@Ag@Pt NPs (**B**).

The principle of the method developed is shown in Figure 1. The Au@Ag@Pt-mAb was first prepared, and the synthesis process of Au@Ag@Pt-mAb is shown in Figure 1A. For the colorimetric detection mode, Au@Ag@Pt-mAb is specifically captured by ACE-BSA immobilized on the T-line when no ACE exists in the sample, leading to color development at the T-line (Figure 1B). At this time, the T-line value read by the colloidal gold reader is the highest, and the test strip forms an obvious visible gray strip. When ACE is present in samples, competitive binding to Au@Ag@Pt-mAb occurs between ACE and ACE-BSA immobilized on the T-line. The color intensity of the T-line decreased and was negatively correlated with ACE concentration. For the photothermal mode, with the increase in ACE concentration, the binding amount of ACE-BSA to Au@Ag@Pt-mAb decreased, and the measured temperature decreased. Based on the relationship between the logarithm of ACE concentration and temperature on the T-line, accurate photothermal quantitative detection was achieved. The two independent detection modes can verify each other‘s results, combining the advantages of rapid screening by colorimetry and high accuracy of photothermal quantification.

### 3.2. Characterization of Au@Ag@Pt NPs

**Figure 2:**
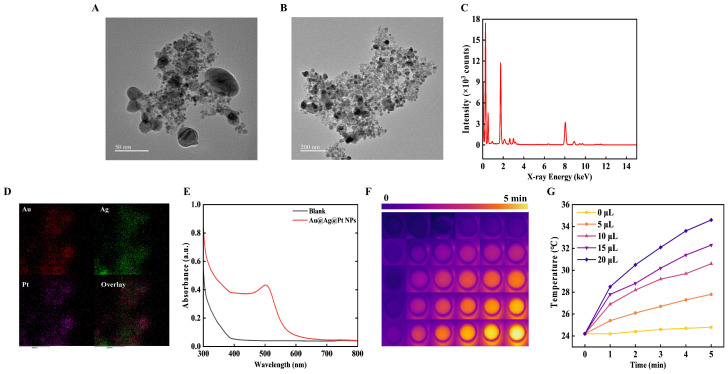
Characterization of Au@Ag@Pt NPs. The TEM images at different scales (**A**,**B**), energy dispersive X-ray spectroscopy spectrum (**C**), element mapping (**D**), UV-Vis spectra of Au@Ag@Pt NPs (**E**), photothermal images (**F**) and the corresponding temperature change curve (**G**) under laser irradiation from 0 to 5 min.

The successful synthesis and complete performance of signal probes are prerequisites for ensuring the sensitivity and stability of DMLFIA. Therefore, the Au@Ag@Pt NPs were systematically characterized from morphology, element distribution, and photothermal properties. The characterization results are shown in Figure 2. As shown in Figure 2A,B, the TEM shows that the Au@Ag@Pt NPs are spherical and the whole is a moderately aggregated cluster structure. This clustered morphology may contribute to enhanced optical properties. At the same time, no serious agglomeration was formed, which did not affect the capillary flow and strip resolution of immunochromatography, and adapted to the detection requirements of test strips. As shown in Figure 2C, the EDS spectrum confirmed the coexistence of Ag, Au and Pt in the nanoparticles. EDS semi-quantitative analysis of the purified Au@Ag@Pt nanoparticles revealed elemental weight fractions of 45.9 wt% Ag, 27.5 wt% Au, and 26.6 wt% Pt (Figure S1). These values are intended to verify the successful incorporation of Ag, Au, and Pt into the trimetallic nanoparticles rather than to represent the theoretical elemental composition calculated from the precursor feeding ratios. Elemental mapping analysis confirmed that Au, Ag, and Pt three elements were uniformly distributed in the nanoparticle system and presented a high degree of overlap, supporting the formation of a trimetallic composite nanoparticle rather than a simple physical mixture of single metal nanoparticles, and the synthesis was successful (Figure 2D). The UV-Vis spectrum of the synthesized Au@Ag@Pt NPs is shown in Figure 2E. The characteristic plasmonic band further confirms the successful formation of the nanoparticles. As shown in Figure 2F, under 808 nm laser irradiation, the nanoparticles presented a significant and stable temperature rise phenomenon. The temperature-time curve of Figure 2G shows that the photothermal mode of the developed DMLFIA has good linear response and high reproducibility, which means that the test strip can not only realize visual and instrumental quantitative detection, but also provide a new idea for photothermal quantitative detection. The above comprehensive characterization results indicated that the prepared Au@Ag@Pt NPs had met the requirements as signal probes for DMLFIA, and laid a solid material foundation for the subsequent establishment of high-performance acetamiprid detection method.

### 3.3. Parameters Optimization of DMLFIA

**Figure 3:**
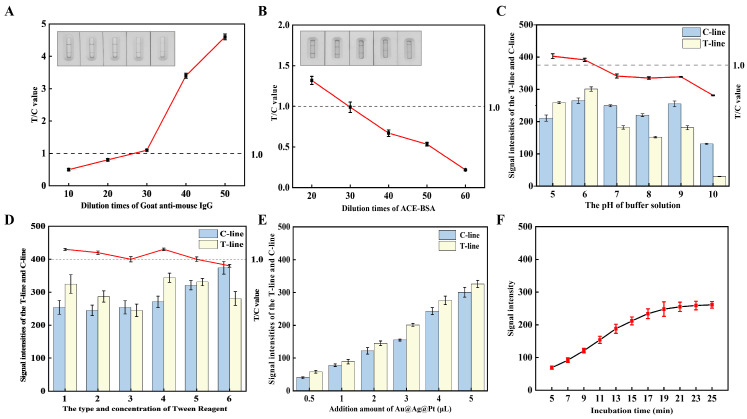
Optimization results for experimental conditions. Optimization of C-line coating concentrations (**A**), T-line coating concentrations (**B**), buffer pH value (**C**), buffer type and concentration (**D**), amount of Au@Ag@Pt-mAb (**E**) and the incubation time (**F**). (**A**,**B**) each contain images of test strips in upper left corner.

The performance of DMLFIA is affected by multiple experimental parameters, including coating concentrations of T-line and C-line, buffer pH, surfactant type and concentration, probe addition amount, and incubation time. Unreasonable parameter settings may lead to poor color rendering, unstable T/C value, large background interference or poor sensitivity. Therefore, in order to achieve the best detection results, this study systematically optimized all the above key parameters, and the optimization results are shown in Figure 3, and the test strips of some optimization conditions are shown in Figure S2.

The coating concentration directly affects the sensitivity and naked eye interpretation of the test strip. If the concentration is too high, it will waste raw materials and may lead to a decrease in sensitivity. If the concentration is too low, the band is not clear and the result cannot be accurately judged. In this experiment, different concentrations of goat anti-mouse IgG antibodies were fixed on the test strip on the C-line, and the same coating antigen was fixed on the T-line. As shown in Figure 3A, after the goat anti-mouse IgG antibody was diluted 30 times, a clear and stable C-line was obtained. Therefore, the dilution of goat anti-mouse IgG antibody was determined to be 30 times. The parameters of C-line were fixed, and the concentration of T-line antigen (Figure 3B) was further optimized. The results showed that when the dilution ratio of ACE-BSA was 30 times, the T/C value was close to 1.0, and the color of T-line and C-line was uniform. Therefore, 30 times diluted ACE-BSA was selected for subsequent experiments. Subsequently, the buffer pH was optimized in the range of 5.0–10.0 (Figure 3C). The buffer solution at pH 6.0 showed the most stable and strongest signal response as the optimal pH condition. In addition, two typical surfactants (Tween-20 and Tween-80) and gradient concentrations (5%, 10%, 20%) were optimized to obtain better experimental results. As shown in Figure 3D, under the condition of 10% Tween-20, the test strip showed clear strips and better visual effects, thus 10% Tween-20 was selected as the optimal surfactant condition for all subsequent experiments. The amount of Au@Ag@Pt-mAb determines the number of binding sites and signal strength. Therefore, the amount of probe added was optimized, and the optimization results are shown in Figure 3E. When the probe volume reached 5.0 μL, the T-line and C-line were clear and stable, the signal was saturated, and there was no obvious background interference. Therefore, 5.0 μL was determined as the optimal probe dosage. Finally, the incubation time within 5–25 min was optimized, and the optimization results are shown in Figure 3F. The signal intensity increased rapidly within 5–20 min and reached a stable plateau after 20 min. Thus, 20 min was selected as the optimal immunoreaction time to balance detection speed and sensitivity.

### 3.4. Sensitivity Analysis of DMLFIA

**Figure 4:**
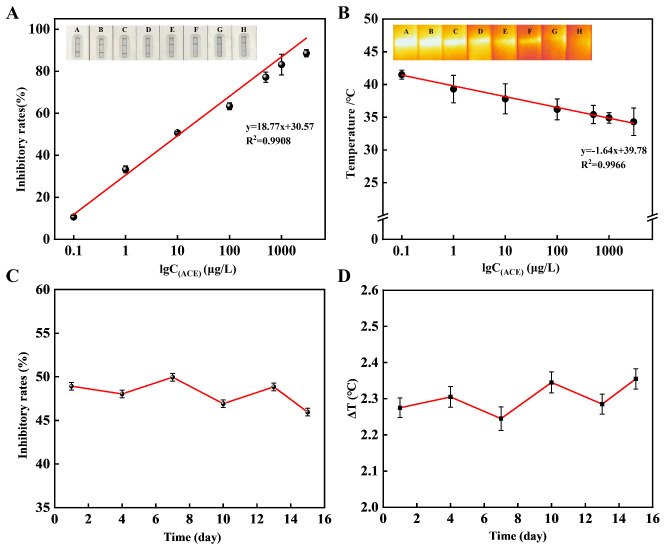
The performance evaluation results of DMLFIA. Standard curve of colorimetric mode (**A**). Standard curve of photothermal mode (**B**). Stability evaluation of colorimetric mode (**C**). Stability evaluation of photothermal mode (**D**). (The upper left corner of (**A**,**B**) are the test strip physical maps of different concentrations of acetamiprid).

Sensitivity is the core index to evaluate the performance of rapid detection strips. Based on the optimal parameters, the sensitivity of the strip was evaluated using a series of ACE standard solutions with gradient concentrations (0, 0.1, 1.0, 10.0, 100.0, 500.0, 1000.0, 3000.0 μg/L). The results are shown in Figure 4. It can be seen from Figure 4A that the visual limit of detection (vLOD) judged by naked eyes was 10.0 μg/L. As shown in Figure 4A, the detection limit of the colorimetric mode after fitting was 0.08 μg/L, the linear equation was y = 18.77x + 30.57, the linear detection range was 0.1–3000 μg/L, and the correlation coefficient (R^2^) was 0.9908, showing a good linear relationship. At the same time, the photothermal signal based on 808 nm laser irradiation also had a good linear response. As shown in Figure 4B, the detection limit of the photothermal mode was 0.1 μg/L, the linear equation was y = 1.64x + 39.78, the linear detection range was 0.1–3000 μg/L, and the correlation coefficient (R^2^) was 0.9966. The developed DMLFIA achieved approximately 174-fold and 139-fold lower LODs than ic-ELISA in colorimetric and photothermal modes, respectively (Figure S3). As shown in Table S2, DMLFIA also exhibits a lower detection limit and a wider linear range than other reported methods. The above advantages prove the feasibility of the established DMLFIA, and also reflect the great application prospect of DMLFIA in the detection of ultra-trace analytes in actual complex samples.

The long-term stability of the constructed DMLFIA was systematically evaluated for both colorimetric and photothermal modes at the concentration of 10.0 μg/L acetamiprid. The results are shown in Figure 4C,D. Prepared test strips were stored under dry conditions at 4 °C, and signal responses were measured every 3 days over a 15-day period, with six replicates performed at each time point. As shown in Figure 4C, in the colorimetric mode, the inhibition rates remained stable at 45.95–49.95%, with standard deviations of 0.40–0.45 and CV below 0.96%. For the photothermal mode, the temperature rise (ΔT) value range is 2.245–2.355 °C, the SD value range is 0.027–0.033, with CV less than 1.45%. These results demonstrate that both detection modes exhibited excellent stability and reproducibility within 15 days.

### 3.5. Specificity Analysis of DMLFIA

Specificity reflects the ability of the strip to specifically recognize the target object without being interfered by other substances. This study selected seven pesticides that were likely to interfere, including imidacloprid, atrazine, dinotefuran, nitenpyram, chlorpyrifos, thiacloprid and trichlorfon, to evaluate the specificity of the strip, and calculated the cross-reactivity (CR) according to the formula in Section 2.6. As shown in Table 1, the specificity evaluation results showed that the cross-reactivity of the strip to all non-target pesticides was lower than 0.39%, and most of them were lower than 0.11%, only imidacloprid with a similar chloropyridyl structure had a slight cross-response, but it was far below the threshold that would cause interference, and would not affect the accurate determination of results. The above results indicated that the strip has excellent specificity and strong anti-interference ability, could specifically recognize acetamiprid, and would not be disturbed by other common pesticides in the actual sample detection, ensuring the accuracy of qualitative and quantitative detection.

### 3.6. Practical Application Results for Plant-Based Food

Accuracy and precision are essential indicators to evaluate the reliability and repeatability of the established analytical method. In this study, accuracy was assessed by the standard addition recovery test, and precision was expressed by the relative standard deviation (RSD). Three concentrations (50.0 μg/L, 100.0 μg/L and 200.0 μg/L) of acetamiprid (ACE) standard solution were added to six kinds of plant-based foods (tangerine peel, lily, fresh corn, soybean, apple and grape). After the optimized pretreatment process, each sample was measured three times in parallel, and the recovery rate and RSD were calculated. At the same time, the test results were compared and verified with the standard HPLC method and the ic-ELISA method to ensure the authenticity and credibility of the test results. The recovery test results of actual samples are shown in Table 2, and the method consistency evaluation results are shown in Figure S4.

As shown in Table 2, the colorimetric mode of DMLFIA yielded satisfactory recoveries ranging from 81.1% to 99.4% with RSD values of 4.6–10.7%, while the photothermal mode provided recoveries of 81.1–92.3% and RSD values of 1.3–6.1%. These results were comparable to those of HPLC (87.0–96.6%, RSD 1.0–2.7%) and ic-ELISA (89.0–98.1%, RSD 2.2–9.2%). All recoveries fell within the acceptable range for pesticide residue analysis, confirming the high accuracy of the proposed method. The RSD values below 11% across all matrices demonstrated excellent intra-assay precision and reproducibility. Notably, as shown in Figure S4, the developed DMLFIA exhibited excellent consistency with both HPLC and ic-ELISA, with no significant differences observed among the three methods (*p* > 0.05). These findings further verified that the established DMLFIA was reliable and accurate for the quantitative determination of ACE residues in complex plant-based food matrices.

## 4. Discussion

In this study, a dual-modal, rapid, and highly sensitive lateral flow immunochromatographic assay was successfully developed for the quantitative detection of acetamiprid (ACE) residues in plant-based foods using Au@Ag@Pt NPs as signal amplification probes. The Au@Ag@Pt NPs were synthesized via a mild sodium citrate reduction method, and its structural, morphological, and photothermal characteristics were fully verified. By systematically optimizing key parameters including coating concentrations of T-/C-lines, the pH of buffer solution, the type and concentration of the Tween Reagent, the additional amount of Au@Ag@Pt-mAb, and the incubation time, the constructed DMLFIA achieved excellent overall performance. The sensor exhibited an ultra-low limit of detection (LOD) of 0.08 μg/L, a wide linear detection range of 0.1–3000 μg/L, and strong specificity with cross-reactivity below 0.39% against non-target pesticides. Spike-recovery tests in six plant-based foods showed favorable recoveries from 81.1% to 99.4% in colorimetric mode and 81.1% to 92.3% in photothermal mode with acceptable precision, and the results were highly consistent with those of HPLC and ic-ELISA, confirming the reliability and accuracy of the proposed method. As shown in Table S2, compared with previously reported analytical systems for ACE detection, the proposed DMLFIA demonstrates significant advantages in both sensitivity and detection range. Traditional instrumental approaches, such as UPLC-MS/MS and FI-CL, report LODs of 3.30 μg/L and 0.148 μg/L, respectively; our method achieves a superior LOD of 0.08 μg/L without relying on bulky, expensive equipment or complex sample preparation. Furthermore, when compared to existing immunoassays, including ic-ELISA (LOD: 0.50 μg/L) and an alternative photothermal Ag@Au LFIA (LOD: 0.12 μg/L), the Au@Ag@Pt-based DMLFIA exhibits enhanced analytical sensitivity. Notably, the linear detection range of our constructed system (0.1–3000 μg/L) is substantially wider than that of conventional ic-ELISA (1.0–150.99 μg/L) and the previously reported Ag@Au LFIA (3.9–250 μg/L), enabling highly versatile quantification across samples with vastly different ACE contamination levels.

The LFIA test strip based on Au@Ag@Pt NPs provides a sensitive, rapid, portable, and low-cost platform for on-site screening of ACE residues, overcoming the limitations of traditional instrumental and immunoassay methods. The dual-mode design combines visual convenience with photothermal accuracy, meeting the requirements of field detection and laboratory confirmation. Although photothermal quantification requires a simple laser device, the method shows great potential for routine monitoring in planting bases, markets, and inspection institutions. Furthermore, the Au@Ag@Pt nanoprobe strategy can be extended to the detection of other pesticide residues, veterinary drugs, and small-molecule contaminants in food and herbal medicines, offering a valuable reference for the development of high-performance rapid immunoassays.

## Figures and Tables

**Table 1:** Results of specificity evaluation.

Variety	Structure	Object Pictures	IC_50_/(μg/L)	CR (%)
Acetamiprid	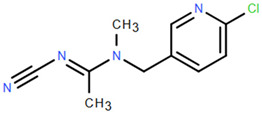	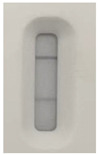	10.85	100.00
Imidacloprid	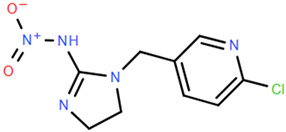	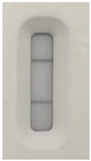	2825.70	<0.39
Atrazine	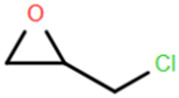	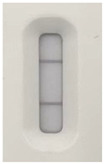	>10,000.00	<0.11
Dinotefuran	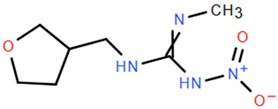	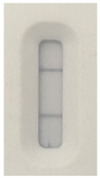	>10,000.00	<0.11
Nitenpyram	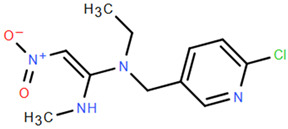	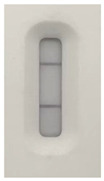	>10,000.00	<0.11
Chlorpyrifos	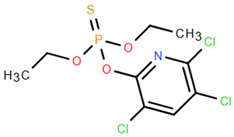	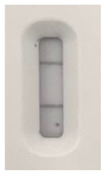	>10,000.00	<0.11
Thiacloprid	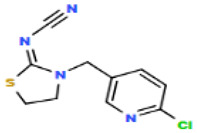	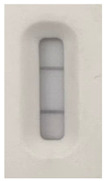	>10,000.00	<0.11
Trichlorfon	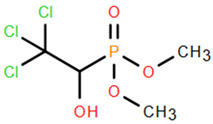	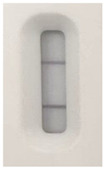	>10,000.00	<0.11

**Table 2:** Comparison of actual sample test results of different methods (*n* = 3).

Samples	Concentration (μg/L)	Colorimetric Mode		Photothermal Mode		HPLC		ic-ELISA	
		Recovery (%)	RSD (%)	Recovery (%)	RSD (%)	Recovery (%)	RSD (%)	Recovery (%)	RSD (%)
Tangerine peel	0	ND ^1^	-	ND	-	-	-	-	-
	50.0	84.6	9.2	81.1	3.5	96.6	2.1	96.0	7.0
	100.0	92.0	7.6	87.8	3.3	91.2	2.4	99.4	6.5
	200.0	88.9	4.6	88.3	2.7	94.8	1.9	90.1	4.9
Lily	0	ND	-	ND	-	-	-	-	-
	50.0	90.6	4.8	83.8	5.4	94.6	1.0	96.4	7.1
	100.0	81.1	8.0	92.1	6.1	91.7	1.2	99.5	4.9
	200.0	84.1	5.5	91.5	5.7	94.4	2.7	95.2	7.3
Fresh corn	0	ND	-	ND	-	-	-	-	-
	50.0	99.4	10.7	89.7	1.3	87.0	1.5	97.2	2.4
	100.0	98.2	9.1	88.7	1.4	88.3	1.7	98.1	9.2
	200.0	92.1	9.4	90.8	2.3	87.1	1.3	89.0	4.5
Soybean	0	ND	-	ND	-	-	-	-	-
	50.0	92.6	9.2	87.4	2.8	96.4	1.4	96.0	2.2
	100.0	87.2	4.6	85.7	3.5	94.2	1.4	95.2	3.2
	200.0	87.2	5.9	92.3	4.0	91.1	2.3	90.6	4.3
Apple	0	ND	-	ND	-	-	-	-	-
	50.0	86.2	7.1	84.5	2.6	92.3	1.8	94.1	5.3
	100.0	88.5	6.3	86.9	3.1	90.5	2.2	97.3	6.8
	200.0	85.7	8.2	89.2	2.9	93.7	1.6	91.8	4.7
Grape	0	ND	-	ND	-	-	-	-	-
	50.0	83.4	9.5	82.7	3.4	89.6	2.0	92.5	7.6
	100.0	87.9	5.8	88.1	2.5	95.1	1.3	96.7	3.9
	200.0	90.3	7.7	91.6	3.7	92.8	2.5	90.2	5.1

^1^ ND: Not Detected.

## Data Availability

The original contributions presented in the study are included in the article/Supplementary Materials, further inquiries can be directed to the corresponding authors.

## Supplementary Materials

The following supporting information can be downloaded at: https://www.mdpi.com/article/10.3390/foods15152598/s1, Figure S1: EDS elemental mapping spectrum of Au@Ag@Pt NPs; Figure S2: The test strips images of buffer pH value (A), buffer type and concentration (B), amount of Au@Ag@Pt-mAb (C); Figure S3: Standard inhibition curve of ic-ELISA; Figure S4: The consistency of DMLFIA with HPLC and ELISA was evaluated; Table S1: Conditions of linear gradient detection for HPLC analysis; Table S2: Comparison of different detection methods for ACE [29,30,31,32,33,34].

## References

[1] Mamy L., Pesce S., Sanchez W., Aviron S., Bedos C., Berny P., Bertrand C., Betoulle S., Charles S., Chaumot A. (2025). Impacts of neonicotinoids on biodiversity: A critical review. Environ. Sci. Pollut. Res..

[2] Stehle S., Ovcharova V., Wolfram J., Bub S., Herrmann L.Z., Petschick L.L., Schulz R. (2023). Neonicotinoid insecticides in global agricultural surface waters-Exposure, risks and regulatory challenges. Sci. Total Environ..

[3] Zuščíková L., Bažány D., Greifová H., Knížatová N., Kováčik A., Lukáč N., Jambor T. (2023). Screening of toxic effects of neonicotinoid insecticides with a focus on acetamiprid: A review. Toxics.

[4] Matsuda K., Ihara M., Sattelle D.B. (2020). Neonicotinoid insecticides: Molecular targets, resistance, and toxicity. Annu. Rev. Pharmacol. Toxicol..

[5] Wang Y., Qin J., Lu Q., Tian J., Ke T., Guo M., Luo J., Yang M. (2023). Residue detection and correlation analysis of multiple neonicotinoid insecticides and their metabolites in edible herbs. Food Chem. X.

[6] Zhang Y., Zhang J., Wang Y., Luo Z., Li X., Wang Y., Luo J., Yang M. (2024). Unveiling the contamination patterns of neonicotinoid insecticides: Detection, distribution, and risk assessment in Panax notoginseng across plant parts. J. Agric. Food Chem..

[7] Sun S., Guo J., Zhu Z., Zhou J. (2024). Microbial degradation mechanisms of the neonicotinoids acetamiprid and flonicamid and the associated toxicity assessments. Front. Microbiol..

[8] Benchikh I., Ziani K., Gonzalez Mateos A., Khaled B.M. (2024). Non-acute exposure of neonicotinoids, health risk assessment, and evidence integration: A systematic review. Crit. Rev. Toxicol..

[9] Hernandez-Jerez A., Coja T., Paparella M., Price A., Henri J., Focks A., Louisse J., Terron A., Binaglia M., Guajardo I.M. (2024). Statement on the toxicological properties and maximum residue levels of acetamiprid and its metabolites. EFSA J..

[10] (2021). National Food Safety Standard—Maximum Residue Limits for Pesticides in Food.

[11] Wang Y., Fu Y., Zhang Y., Zhao Z., Xu T., Chen Y., Luo J., Yang M. (2023). Residue, distribution and degradation of neonicotinoids and their metabolites in chrysanthemum plants and cultivated soils. Microchem. J..

[12] Wang Y., Fu Y., Wang Y., Lu Q., Ruan H., Luo J., Yang M. (2022). A comprehensive review on the pretreatment and detection methods of neonicotinoid insecticides in food and environmental samples. Food Chem. X.

[13] Zhang J., Liu J., Wang Y., Wang Y., Yang R., Zhou X. (2023). Simultaneous determination of ten neonicotinoid insecticides and a metabolite in human whole blood by QuEChERS coupled with UPLC-Q Exactive orbitrapHigh-resolution mass spectrometry. J. Chromatogr. B.

[14] Almenhali A.Z., Eissa S. (2024). Aptamer-based biosensors for the detection of neonicotinoid insecticides in environmental samples: A systematic review. Talanta.

[15] Chakraborty S. (2024). Democratizing nucleic acid-based molecular diagnostic tests for infectious diseases at resource-limited settings—From point of care to extreme point of care. Sens. Diagn..

[16] Bai T., Wang L., Wang M., Zhu Y., Li W., Guo Z., Zhang Y. (2022). Strategic synthesis of trimetallic Au@Ag-Pt nanorattles for ultrasensitive colorimetric detection in lateral flow immunoassay. Biosens. Bioelectron..

[17] Jiao C., Duan W., Wu X., Shang Y., Zhang F., Zhang M., Chen X., Zeng J., Yang C. (2023). Multifunctional nanoprobes-amplified enzyme-linked immunosorbent assay on capillary: A universal platform for simple, rapid, and ultrasensitive dual-mode pathogen detection. Anal. Chem..

[18] He X., Hao T., Geng H., Li S., Ran C., Huo M., Shen Y. (2023). Sensitization strategies of lateral flow immunochromatography for gold modified nanomaterials in biosensor development. Int. J. Nanomed..

[19] Yin X.C., Liu S.J., Kukkar D., Wang J.L., Zhang D.H., Kim K.H. (2024). Performance Enhancement of the Lateral Flow Immunoassay by Use of Composite Nanoparticles as Signal Labels. TrAC Trends Anal. Chem..

[20] Cho Y., Choi Y., Jang Y., Seong H. (2025). Nanomaterial-enhanced biosensing: Mechanisms and emerging applications. Adv. Healthc. Mater..

[21] Li X., Fu Y., Lin Y., Du D. (2026). Emerging 2D materials and their hybrid nanostructures for label-free optical biosensing: Recent progress and outlook. Adv. Funct. Mater..

[22] Xu M., Nahar L., Ritchie K.J., Wang C., Cheng L., Wu Z., Sarker S.D., Guo M. (2026). Recent advances in nanomaterial-based optical biosensors and their biomedical and biopharmaceutical applications. J. Pharm. Anal..

[23] Chicea D., Nicolae-Maranciuc A. (2025). Metal nanocomposites as biosensors for biological fluids analysis. Materials.

[24] Andryskova P., Prucek R. (2026). Multimetallic nanostructures: Innovations and applications in environmental remediation, biosensing, and medical technologies. Nanoscale.

[25] Gilroy K.D., Ruditskiy A., Peng H.C., Qin D., Xia Y. (2016). Bimetallic nanocrystals: Syntheses, properties, and applications. Chem. Rev..

[26] Huang X., Yang Y., Zhou H., Hu L., Yang A., Jin H., Zheng B., Pi J., Xu J., Sun P. (2025). Coupling of an Au@AgPt nanozyme array with a micrococcal nuclease-specific responsiveness strategy for colorimetric/SERS sensing of Staphylococcus aureus in patients with sepsis. J. Pharm. Anal..

[27] Lee H.J., Hanyu D., Dao A.T.N., Kasai H., Suzuki M., Yabu H., Nakatani H., Kaneko K. (2024). Controlling the Composition and Nanostructure of Au@Ag-Pt Core@Multi-Shell Nanoparticles Prepared by Co-Reduction Method. Mater. Today Chem..

[28] Huang X., Chen L., Zhi W., Zeng R., Ji G., Cai H., Xu J., Wang J., Chen S., Tang Y. (2023). Urchin-shaped Au-Ag@Pt sensor integrated lateral flow immunoassay for multimodal detection and specific discrimination of clinical multiple bacterial infections. Anal. Chem..

[29] Biswas S., Mondal R., Mukherjee A., Sarkar M., Kole R.K. (2019). Simultaneous determination and risk assessment of fipronil and its metabolites in sugarcane, using GC-ECD and confirmation by GC-MS/MS. Food Chem..

[30] Jin D., Xu Q., Yu L., Mao A., Hu X. (2016). A novel sensor for the detection of acetamiprid in vegetables based on its photocatalytic degradation compound. Food Chem..

[31] Zhang D., He M., Qin C., Wu Z., Cao M., Ni D., Yu Z., Liang P. (2024). A highly effective SERS platform formed by the fabrication of Ag@ZIF-8@Au nanoparticles for rapid detection of acetamiprid in environment. Spectrochim. Acta Part A.

[32] Zhou T., Zhang B., Xie X., Liu Y., Li H., Jin H., Lin Y., Wei F., Wang Y. (2025). Construction and Application of Indirect Competitive Enzyme-Linked Immunosorbent Assay for Acetamiprid in Traditional Chinese Medicine. Toxics.

[33] Zhu Z., Guan L., Zhang Y., Wu J., Feng J., Dong S. (2026). A dual-mode lateral flow immunoassay utilizing Ag@Au nanoparticles and aptamers enables colorimetric and photothermal detection of acetamiprid. Talanta.

[34] Nisa K.U., Kakar A.U.R., Asghar M., Samiullah, Naqeebullah, Kakar M.Q., Hakeem A., Rehman H.U. (2025). Photoinduced chemiluminescence method for the determination of acetamiprid and pendimethalin with flow injection analysis in fruit and water samples. J. Food Compos. Anal..

