# Supplementary material for: Au@Ag@Pt Trimetallic Nanoparticle-Enhanced Dual-Mode Lateral Flow Immunochromatographic Assay for Ultrasensitive and Rapid Detection of Acetamiprid in Plant-Based Foods
Source: Foods. 2026 Jul 24;15(15):2598. doi: 10.3390/foods15152598 (PMC13465176; doi:10.3390/foods15152598)
Supplement: Supplementary file 1 [file foods-15-02598-s001.zip › foods-4388407-supplementary.pdf]

Article

Au@Ag@Pt Trimetallic Nanoparticle-Enhanced Dual-Mode Lateral Flow Immunochromatographic Assay for Ultrasensitive and Rapid Detection of Acetamiprid in Plant-Based Foods

Qingqing Li [1], Xuecheng Zhang [1], Shurun Chen [1], Bowen Guo [1], Qingwen Cao [2], Xinyue Feng [1], Xiaoping Yu [1], Xiaoyu Jia [3], Biao Zhang [1,*] and Hongqing Wang [2,*]

[1] Key Laboratory of Microbiological Metrology, Measurement & Bio-Product Quality Security, State Administration for Market Regulation, College of Life Sciences, China Jiliang University, Hangzhou 310018, China; liqingqing7295@163.com (Q.L.); 2200901111@cjlu.edu.cn (X.Z.); 19057243957@139.com (S.C.); guobowen2024@163.com (B.G.); fxy18767053369@163.com (X.F.); yxp@cjlu.edu.cn (X.Y.)

[2] Hangzhou Institute of Food and Drug Control, Hangzhou 310022, China; cqw891002@hotmail.com

[3] Institute of Agricultural Products Preservation and Processing Science and Technology, Academy of Agricultural Sciences, Tianjin 300384, China; jiaxiaoyu331@163.com

* Correspondence: zhangbiao9129@163.com (B.Z.); cairouer0524@126.com (H.W.)

Contents

Supplementary experimental methods
Figure S1 Figure S2 Figure S3 Figure S4
Table S1 Table S2

Development of Enzyme-linked immunosorbent assay (ELISA)

The detailed experimental protocols for the ACE-based enzyme-linked immunosorbent assay (ELISA) are described below. A 96-well microplate was coated with ACE coating antigen solution at a volume of 100.0 μL per well (containing 0.05 μg coating antigen per well), followed by overnight incubation at 4 °C to achieve stable antigen immobilization. After three washes with 0.01 mol/L PBST buffer supplemented with 10% Tween-20, 200.0 μL of 1% skim milk blocking buffer was dispensed into each well, and a 1.5 h incubation was performed at 37 °C for the inhibition of non-specific binding. Upon the completion of three additional washing cycles, equal volumes (50.0 μL) of ACE standard solutions or testing samples and 8000-fold diluted primary ACE antibody (prepared in 0.01 mol/L PBS) were sequentially supplemented into individual wells. A 1.5 h incubation at 37 °C was implemented to facilitate adequate antigen-antibody conjugation.After four repeated washing steps, 100.0 μL of 400-fold diluted HRP-conjugated secondary antibody was added to each well, and the microplate was incubated at 37 °C for 40 min. Subsequent to sufficient washing procedures, 100 μL of freshly prepared TMB chromogenic substrate solution was loaded into each well, and the chromogenic reaction was conducted at 37 °C for 12 min under dark incubation conditions. Thereafter, 50 μL of 1.25 mol/L sulfuric acid termination solution was injected to instantly quench the chromogenic reaction. Finally, the absorbance of each well was measured at a wavelength of 450 nm using a Thermo Varioskan LUX multimode microplate reader.

HPLC reference conditions are as follows:

a) C_{18} reversed-phase column (column length 250 mm, inner diameter 4.6 mm, particle size 5 μm), linear gradient for HPLC analysis is shown in Table S1;

b) Flow rate: 0.7 mL/min

c) Column temperature: 30.0 °C

d) Injection volume: 20.0 μL

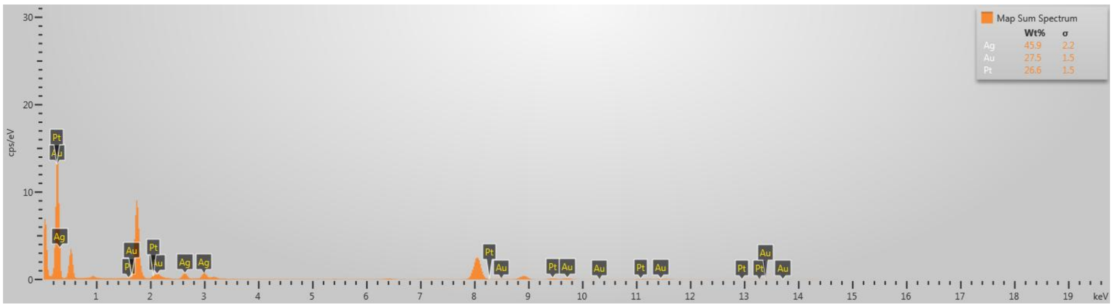

Figure S1. EDS elemental mapping spectrum of Au@Ag@Pt NPs.

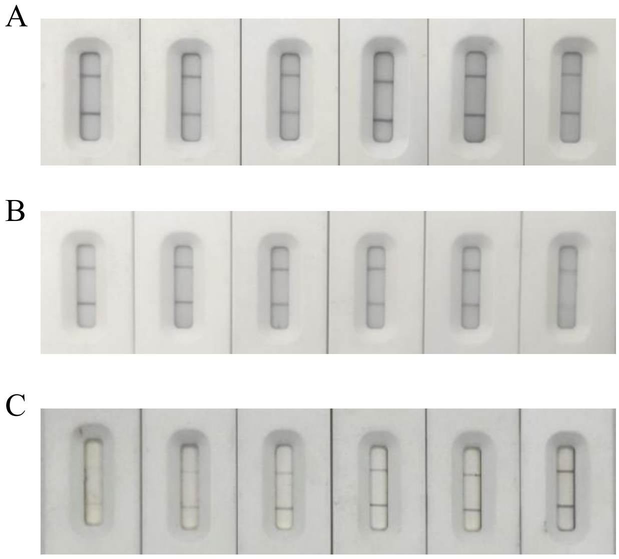

Figure S2. The test strips images of buffer pH value (A), buffer type and concentration (B), amount of Au@Ag@Pt-mAb (C).

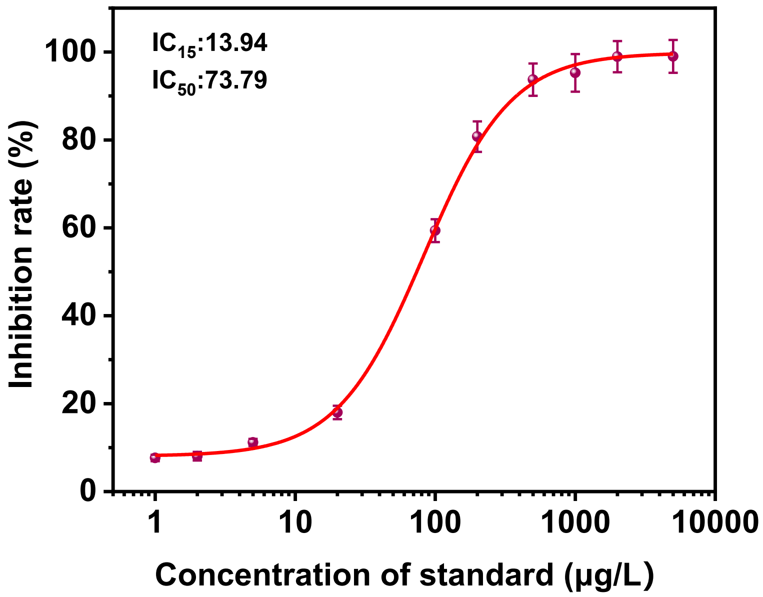

Figure S3. Standard inhibition curve of ic-ELISA.

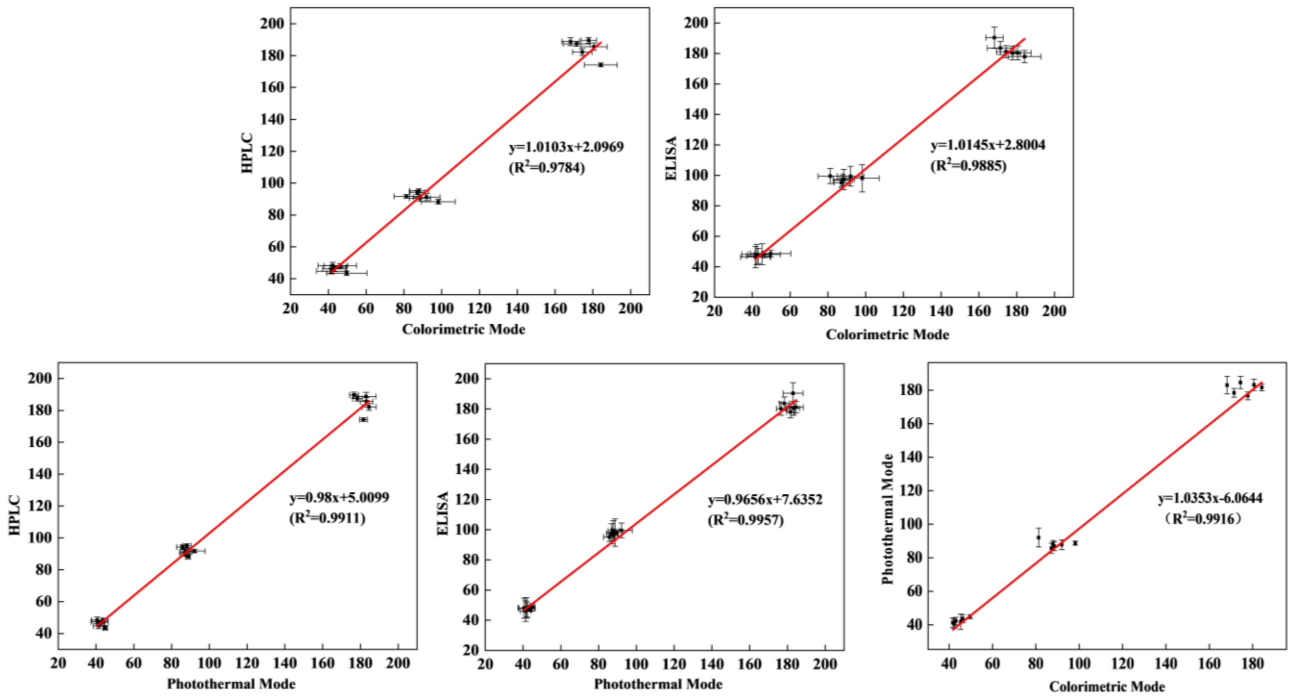

Figure S4. The consistency of DMLFIA with HPLC and ELISA was evaluated.

Table S1. Conditions of linear gradient detection for HPLC analysis.

Time (min)	A: Water (%)	B: Methanol (%)
0	70	30
3	70	30
7	40	60
9	10	90
11	10	90
12	70	30

Table S2. Comparison of different detection methods for ACE [29–34].

Number	Method	Detection limit(μg/L)	Linear Range (μg/L)	Recovery Range (%)	Sample	Reference
1	UPLC-MS/MS	3.30	None	80.7-98.5	Soil, sugarcane	[29]
2	PD-DPV	0.0445	2.23-445.00	80.0-105.0	Cucumber, cabbage	[30]
3	SERS	0.198	0.22-22267	85.0-110.0	Lake water, tea leaves, oranges	[31]
4	ic-ELISA	0.50	1.0-150.99	86.9-104.8	TCMs	[32]
5	Ag@Au LFIA (Phototherrmal)	0.1155	3.9-250	87.0-113.0	Cucumber, cabbage	[33]
6	FI-CL	0.148	0.1-1000	76.4-104.5	Fruits, water	[34]
7	Au@Ag@Pt	0.08	0.1-3000	81.1-99.4	Agricultural products	Our work

References

29. Biswas, S.; Mondal, R.; Mukherjee, A.; Sarkar, M.; Kole, R.K. Simultaneous determination and risk assessment of fipronil and its metabolites in sugarcane, using GC-ECD and confirmation by GC-MS/MS. *Food Chem.* **2019**, *272*, 559–567. https://doi.org/10.1016/j.foodchem.2018.08.087.
30. Jin, D.; Xu, Q.; Yu, L.; Mao, A.; Hu, X. A novel sensor for the detection of acetamiprid in vegetables based on its photocatalytic degradation compound. *Food Chem.* **2016**, *194*, 959–965. https://doi.org/10.1016/j.foodchem.2015.08.118.
31. Zhang, D.; He, M.; Qin, C.; Wu, Z.; Cao, M.; Ni, D.; Yu, Z.; Liang, P. A highly effective SERS platform formed by the fabrication of Ag@ZIF-8@Au nanoparticles for rapid detection of acetamiprid in environment. *Spectrochim. Acta Part A* **2024**, *308*, 123754. https://doi.org/10.1016/j.saa.2023.123754.
32. Zhou, T.; Zhang, B.; Xie, X.; Liu, Y.; Li, H.; Jin, H.; Lin, Y.; Wei, F.; Wang, Y. Construction and Application of Indirect Competitive Enzyme-Linked Immunosorbent Assay for Acetamiprid in Traditional Chinese Medicine. *Toxics* **2025**, *13*, 982. https://doi.org/10.3390/toxics13110982.
33. Zhu, Z.; Guan, L.; Zhang, Y.; Wu, J.; Feng, J.; Dong, S. A dual-mode lateral flow immunoassay utilizing Ag@Au nanoparticles and aptamers enables colorimetric and photothermal detection of acetamiprid. *Talanta* **2026**, *296*, 128508. https://doi.org/10.1016/j.talanta.2025.128508.
34. Nisa, K.U.; Kakar, A.U.R.; Asghar, M.; Samiullah; Naqeebullah; Kakar, M.Q.; Hakeem, A.; Rehman, H.U. Photoinduced chemiluminescence method for the determination of acetamiprid and pendimethalin with flow injection analysis in fruit and water samples. *J. Food Compos. Anal.* **2025**, *147*, 108069. https://doi.org/10.1016/j.jfca.2025.108069.

Disclaimer/Publisher's Note: The statements, opinions and data contained in all publications are solely those of the individual author(s) and contributor(s) and not of MDPI and/or the editor(s). MDPI and/or the editor(s) disclaim responsibility for any injury to people or property resulting from any ideas, methods, instructions or products referred to in the content.
